# The effect of lyophilization and storage time on the survival rate and hydrolytic activity of *Trichoderma* strains

**DOI:** 10.1007/s12223-017-0581-0

**Published:** 2018-01-05

**Authors:** Monika Grzegorczyk, Anna Kancelista, Wojciech Łaba, Michał Piegza, Danuta Witkowska

**Affiliations:** Department of Biotechnology and Food Microbiology, Wrocław University of Environmental and Life Sciences, ul. Chełmońskiego 37, 51-630 Wrocław, Poland

## Abstract

The study evaluates the survivability and storage stability of seven *Trichoderma* strains belonging to the species: *T. harzianum* (1), *T. atroviride* (4), and *T. virens* (2) after the lyophilization of their solid state cultures on wheat straw. Biomass of *Trichoderma* strains was freeze-dried with and without the addition of maltodextrin. Furthermore, in order to determine the ability of tested *Trichoderma* strains to preserve selected technological features, the biosynthesis of extracellular hydrolases (cellulases, xylanases, and polygalacturonases) after a 3-month storage of lyophilizates was investigated. Strains of *T. atroviride* (except TRS40) and *T. harzianum* TRS85 showed the highest viability after lyophilization process (up to 100%). After 3 months of storage, *T. atroviride* TRS14 exhibited the highest stability (95.23%); however, the number of active conidia remained at high level of 10^6^–10^7^ cfu/g for all tested *T. atroviride* strains and *T. harzianum* TRS85. Interestingly, after a 3-month storage of lyophilized formulations, most of the tested *Trichoderma* strains exhibited higher cellulolytic and xylanolytic activities compared to the control, i.e., before freeze-drying process. The highest activities of these enzymes exhibited the following: *T. atroviride* TRS14–2.37 U/g and *T. atroviride* TRS25–21.47 U/g, respectively, whereas pectinolytic activity was weak for all tested strains, with the highest value of 0.64 U/g registered for *T. virens* TRS109.

## Introduction

Fungi of the genus *Trichoderma*, soil-borne plant symbionts, located in seeds, stems, and roots (Pandya et al. 2011), are recognized for their antagonistic activity against some phytopathogenic fungi and supportive effect on plant growth (Benítez et al. 2004; Druzhinina et al. 2010). These fungi occur in all climate zones due to their remarkable adaptation to various environmental conditions (Benítez et al. 2004). Commercially, *Trichoderma* are mainly used in crop protection, as components of biological control agents (BCAs) (Witkowska and Maj 2002; Piegza et al. 2009; Grzegorczyk et al. 2015) and in microbial enzyme production (cellulases, xylanases, polygalacturonases, and others) (Soliman et al. 2012), further used in food processing, brewing and wine industries (Ganga et al. 1997; Bai et al. 2008), biofuel production (Kovacs et al. 2009; Gusakov 2011), paper and textile industries (Kuhad et al. 2011), and in the management of waste and residues from agriculture and agro-food industries (Kancelista et al. 2013, Phitsuwan et al. 2013; Smolińska et al. 2014).

Solid state fermentation (SSF) with the use of agricultural waste materials is a technique used for the production of fungal biomass and enzymes, which generates lower costs and pollution, while maintaining high biomass yield and enzyme production, in comparison to submerged fermentation (SmF) (Pandey 1991; Sukumaran et al. 2009). Agricultural wastes are also considered significant potential for the development of biofuel production. Hydrolytic demolition by lignocellulosic enzymes is one of the most studied approach (Masutti et al. 2012). Delignification process was investigated on straw SSF with *Trametes versicolor* and *Pleurotus ostreatus* (Valmaseda et al. 1991), where *P. ostreatus* showed better results than the other tested fungus in xylanase and exocellulase production. Also, *Aspergillus niger* produced high yields of xylanases on wheat straw in the studies conducted by Sanghvi et al. (2010). These encouraging results prompted researchers to conduct further trials, also on fungi from the genus *Trichoderma*. Therefore, the production of lignocellulosic enzymes in SSF cultures, with the use of agricultural and agro-industrial residues, such as wheat bran, corn stover, sugar cane bagasse, beet molasses, and citrus fruit peels have been extensively studied also using *Trichoderma* strains, isolated from different environments, for their abovementioned valuable features in many sectors of agro-industry (Soliman et al. 2012; Mohamed et al. 2013).

High enzymatic activities made them applicable in many industries and innovative agriculture, but also in utilization and composting of waste from households or agricultural and food industries, comprised mainly of cellulose, hemicellulose, and pectin as well as in in the improvement of nutrient digestibility in animal diets (Kuhad et al. 2011).

In order to manufacture biological formulations, cells, spores, or other biological compounds must first undergo a preservation process to induce dormancy and provide stability of the product in prolonged storage. Among several, based on lowering of water availability, methods of preservation, such as freezing, drying, or freeze-drying, the latter seems to be the most beneficial for the retainment of viability of microorganisms and their distinctive functionality (Day and Stacey 2007). Freeze-dried microorganisms are enclosed in glassy structure, which is very beneficial for long-term storage of dehydrated microorganisms, due to the immobilization of molecules in the disordered structure, which in turn protects cell membrane and proteins from damage. Lyophilized cultures can be stored and transported at room temperature (Day and Stacey 2007). The addition of cryoprotectants is considered to be beneficial during freeze-drying process. For instance, lactose minimizes changes in the cell membrane during dehydration, by replacing the water molecules. Other saccharides, such as trehalose or maltodextrin, protect against denaturation of proteins (Tan et al. 2007). Stability of microorganisms during storage is reported to be influenced by drying process, storage conditions, and rehydration processes (Daryaei et al. 2016). Several stress factors, such as temperature, pH, nutritional status, water activity, and relative humidity (RH) may affect the viability and biological properties of fungal conidia (Magan 2001; Texido et al. 2005; Daryaei et al. 2016).

The aim of this study was to preserve the biomass of polish *Trichoderma* strains in a freeze-drying process on wheat straw and to assess their survival and abilities for biosynthesis of selected hydrolases (cellulases, xylanases, and polygalacturonases), after 3 months of storage of lyophilizates.

## Materials and methods

### Fungal strains

Seven strains, used in this study, *T. atroviride* TRS14, *T. atroviride* TRS25, *T. atroviride* TRS40, *T. atroviride* TRS43, *T. harzianum* TRS85, and two strains of the species *T. virens*: TRS106 and TRS109, were obtained from the culture collection at the Research Institute of Horticulture in Skierniewice (Poland) and they were selected based on the innovative AMOR system, defining the usefulness of *Trichoderma* strains in terms of their antagonistic activity against phytopathogenic fungi (Szczech et al. 2011 - Patent Application no. P.397659). Fungal strains TRS14, TRS25, TRS85, TRS106, and TRS109 were isolated from champignon farm (Szczech et al. 2008; Skoneczny et al. 2015), whereas TRS43 was isolated from forest soil in the National Park of Wielkopolska (Poland) and TRS40—from compost in Rzeczyce (Poland). All of the isolated strains were subsequently cultured in potato dextrose broth (PDB), followed by cultivation in potato dextrose agar (PDA) for several generations and stored in − 80 °C as follows: two discs (5-mm diameter) were bored out of an agar plate together with the fungal colony growing on it and placed into cryotubes, containing 1 mL of 10% (*v*/*v*) glycerol-water solvent.

### Culture conditions

#### Recovery of fungal cultures

Discs of PDA with fungal biomass of each *Trichoderma* strain, obtained from the − 80 °C, were transferred onto PDA medium and cultivated for 7–10 days at the temperature of 25 °C. Subsequently, biomass was harvested and inoculated into PDB liquid medium and cultivated for 7 days at the temperature of 25 °C, shaken (140 rpm), followed by reinoculation of the obtained fungal biomass onto PDA medium again. After 7 days of its incubation at 25 °C, conidia were collected, using 10 mL of 0.1% Tween 80 solution. The obtained suspension of fresh conidia served as inoculum for PDA cultures in Kolle flasks.

#### Conidia production

*Trichoderma* strains were cultivated in 400-mL Kolle flasks, containing PDA medium (24 g/L) at 25 °C for 7 days. After incubation, conidia were collected using 10 mL of 0.1% Tween 80 solution. The resulting suspensions of conidia were standardized in Thoma chamber and used as inoculum in SSF cultures.

#### Solid state fermentation cultures

Wheat straw, a waste lignocellulosic material, obtained from the Department of Crop Production (University of Environmental and Life Sciences in Wrocław, Poland) was used as medium in SSF cultures of tested *Trichoderma* strains. Forty grams of shredded into 2–3-cm pieces of wheat straw was transferred into 1000-mL Roux flasks, wherein tap water was added to obtain a moisture content of 65–75%. SSF medium, after thorough mixing, was sterilized twice at an interval of 24 h and then inoculated with, as described above, standardized suspension of conidia (5 × 10^6^ conidia/g of culture medium in the final volume of 20 mL). Then, cultures were incubated in a phytotron chamber (binder) at 25 °C, humidity of 75%, and 24-h lighting for 240 h. Every 2 days, the cultures were hand-agitated in order to improve aerobic conditions for growing *Trichoderma* strains.

#### Determination of *Trichoderma* strains’ capability for biosynthesis of hydrolytic enzymes

In order to determine the abilities of all tested *Trichoderma* strains for the biosynthesis of cellulases, xylanases and polygalacturonases, 250-mL Erlenmeyer flasks, containing 10 g of SSF were individually inoculated with lyophilizates of conidia, after 3 months of their storage at room temperature or with fresh, not preserved by freeze-drying, conidial suspensions (control), in the amount of 1% of the medium. SSF cultures of *Trichoderma* strains were carried out as already described for 240 h.

### Preservation of biomass in the freeze-drying process

The culture medium (wheat straw) overgrown with mycelium and conidia was preserved by lyophilization. Fifty grams of each *Trichoderma* culture was transferred into a 500-mL round bottom flask together with 50 mL of a 20% sterile solution of maltodextrin (Malt) as a shielding agent (final concentration of cryoprotectant—10%) or an equivalent amount of sterile distilled water (control), thoroughly mixed, and subsequently subjected to freeze-drying process, preceded by external freezing to temperature of − 26 °C. Freeze-drying was performed with the Labconco triad lyophilizer, at an external manifold a pressure of 0.2 mbar for 20 h. After preservation, lyophilizates were transferred into plastic bags, vacuum sealed, and stored at room temperature for 3 months.

### Analytical methods

#### Microbiological methods

Determination of the number of viable cells (cfu/g) after SSF cultures and in the preserved biopreparations, as well as in the stored lyophilizates of *Trichoderma*, was performed using Koch’s dilution plate method, on a PDA medium with the addition of Bengal rose (0.035 g/L). Three consecutive dilutions of conidial suspensions were plated out in triplicate. The number of cfu/g was determined and the percentage of viable cells (% survivability) was expressed as a percentage of surviving cells compared to live cells prior to preservation. Similarly, the storage stability (%) was defined as a percentage of surviving cells after a 3-month storage relative to number of live cells after freeze-drying process.

#### Biochemical methods

Preparation of samples for enzymatic assays such as contents of flasks after culture was extracted with 0.1% Tween 80 for 30 min at 160 rpm (VWR Advances Digital Shaker) at room temperature. Then, the obtained post-culture suspensions, containing extracellular enzymatic proteins, were centrifuged (10,000 rpm and 4 °C for 10 min) and the resulting supernatants were subjected to enzymatic activity assays. The enzymatic activities of cellulases, xylanases (Witkowska et al. 1997), and polygalacturonases (Witkowska and Bień 1991), in extracts from SSF cultures of *Trichoderma* strains, were determined according to the methods described in the abovementioned literature, using DNS reagent for the determination of reducing compounds, i.e., the products released by the enzymatic reaction (Miller 1959). The unit of enzyme activity was expressed as the amount of enzyme which liberates 1 μmol of reducing sugars per minute per 1 g of culture medium (U/g).

#### Water activity, moisture content, and pH analysis

In all biological materials, i.e., in samples after SSF cultures, after freeze-drying of SSF cultures, and after a 3-month storage of lyophilizates, water activity (*a*_w_), moisture content (%), and pH value were determined, using the following: Decagon Aqualab Devices Series 3, Radwag MAC 110/NH, and pH METER CP-505 (Elmetron), respectively. All analyses were performed in duplicate.

### Statistical analysis

Data from individual experiments (number of viable cells and enzymatic activity) were analyzed separately using the Statistica package software (Version 12; Statsoft Inc). The significance of differences between mean values of cfu/g and U/g were assessed in one-way analysis of variance, according to LSD Fisher’s test at significance level of *p* < 0.05.

## Results

### Fungal biomass yield in SSF cultures on wheat straw

All of the tested *Trichoderma* strains, cultivated in solid state fermentation (SSF) cultures on wheat straw, were tested for their viability referred to as cfu/g. Biomass yield of 10-day SSF *Trichoderma* cultures, inoculated with fresh (non-preserved) conidia, differed within tested strains. Among all *Trichoderma* isolates, the biomass yield registered for *T. harzianum* TRS85 was over 7 × 10^8^ cfu/g, followed by *T. atroviride* strains, where the number of viable cells ranged from less than 7 × 10^7^ (TRS40) to over than 2 × 10^8^ (TRS25) cfu/g. In turn, lower number of viable cells was registered for *T. virens* TRS106 and TRS109, as compared to all tested *Trichoderma* isolates (Table 1). After incubation period, the moisture content and water activity were 68% and 1.0, respectively, which is desirable for SSF cultures (Table 2).

**Table 1:** Number of viable cells, expressed as cfu/g, after cultivation of *Trichoderma* strains in SSF cultures, after lyophilization process of SSF cultures, and after the 3-month storage of lyophilizates

Strain		cfu/g				
		SSF culture	After lyophilization		After storage	
			Water	Maltodextrin	Water	Maltodextrin
*T. atroviride*	TRS14	1.13 × 10^8^ a	1.13 × 10^8^ a	9.12 × 10^7^ ab	3.11 × 10^7^ b	8.69 × 10^7^ ab
*T. atroviride*	TRS25	2.17 × 10^8^ a	2.17 × 10^8^ a	2.12 × 10^8^ a	2*.*35 × 10^6^ b	1.42 × 10^6^ b
*T. atroviride*	TRS40	6.75 × 10^7^ a	1.04 × 10^7^ ab	1.82 × 10^7^ ab	4.39 × 10^6^ b	1.31 × 10^7^ ab
*T. atroviride*	TRS43	1.31 × 10^8^ a	1*.*31 × 10^8^ a	1.18 × 10^8^ a	3.45 × 10^7^ b	3*.*54 × 10^7^ b
*T. harzianum*	TRS85	7.47 × 10^8^ a	7.47 × 10^8^ a	5.90 × 10^8^ b	5.80 × 10^7^ c	5.81 × 10^7^ c
*T. virens*	TRS106	8.04 × 10^6^ a	2*.*03 × 10^6^ b	9.93 × 10^5^ b	1.58 × 10^3^ b	2.86 × 10^4^ b
*T. virens*	TRS109	5.10 × 10^6^ a	2*.*95 × 10^5^ ab	1.68 × 10^5^ bc	1*.*00 × 10^4^ c	1*.*00 × 10^4^ c

Data presented as mean of cfu/g. In each row, values followed by different letters are significantly different according to LSD Fisher’s test (*p* < 0.05)

**Table 2:** Moisture content, water activity, and pH in SSF control cultures and in lyophilizates of *Trichoderma* strains right after lyophilization and the 3-month storage

	Moisture content (%)	*a*_w_	pH
SSF—control	68.49 ± 1.70	1.00 ± 0.00	8.05 ± 0.14
Lyophilizates—0 month	3.14 ± 0.14	0.09 ± 0.08	7.90 ± 0.26
Lyophilizates—3 months	4.95 ± 0.53	0.21 ± 0.03	7.82 ± 0.27

Data presented as mean values ± standard error

### Survivability and storage stability of *Trichoderma* strains after their preservation by lyophilization

**Fig. 1:**
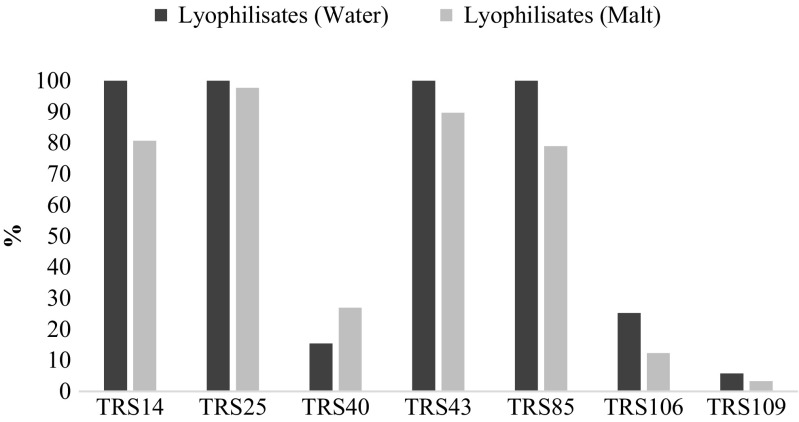
Survivability of *Trichoderma* strains in lyophilizates with the addition of maltodextrin (malt) or distilled water (water) right after lyophilization process

*Trichoderma* strains were tested for their viability after lyophilization process and after a 3-month storage of lyophilizates, as mentioned above. Moreover, respectively, the survivability and storage stability were determined. In general, higher survivability was registered for all tested *Trichoderma* strains (except *T. atroviride* TRS40) in control lyophilizates, with the addition of distilled water, as compared to bioformulation added with maltodextrin. In detail, lyophilization process did not affect the number of viable cells of *T. atroviride* TRS25, TRS43, TRS14, and *T. harzianum* TRS85 in control lyophilizates (100% survivability), whereas in lyophilizates added with maltodextrin, the survivability registered for these strains ranged from 78 to 97% (Fig. 1). Significantly lower number of cells was obtained for *T. atroviride* TRS40 and *T. virens* strains after freeze-drying, regardless of the addition of cryoprotectant, as compared to the control SSF cultures, inoculated with fresh conidia (Fig. 1). The lowest survivability was registered for *T. virens* TRS109 in lyophilizates with maltodextrin (Fig. 1), which corresponded to 10^5^ cfu/g (Table 1). Moisture content and water activity in lyophilizates decreased which is desirable after freeze-drying process. The pH value of freeze-dried SSF cultures lowered, comparing to non-preserved cultures; however, it remained slightly alkaline (Table 2).

**Fig. 2:**
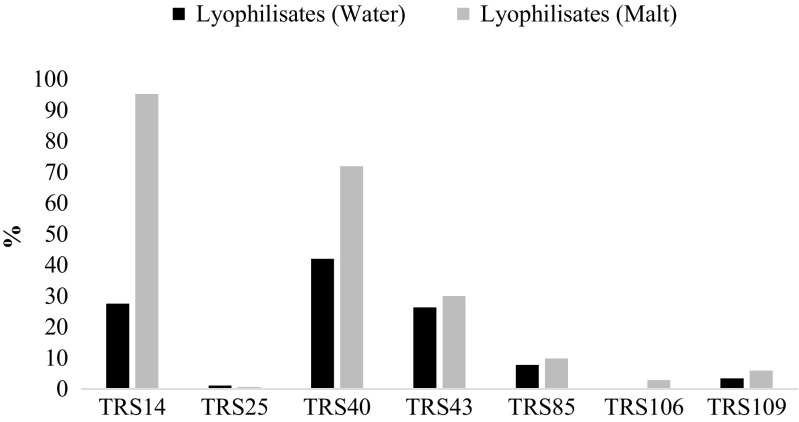
Storage stability of *Trichoderma* strains in lyophilizates with the addition of maltodextrin (malt) or distilled water (water), after the 3-month storage

After storage of *Trichoderma* lyophilizates, the number of viable cells decreased; however, in general, higher storage stability was registered for all tested *Trichoderma* strains (except *T. atroviride* TRS25) in lyophilizates added with maltodextrin, as compared to control lyophilizates. The highest values of storage stability were obtained for *T. atroviride* TRS14 and TRS40—95.23 and 71.90%, respectively (Fig. 2), which corresponded to 10^7^ cfu/g (Table 1). Although the storage stability in lyophilizates, regardless of the addition of cryoprotectant, were of the low values for *T. atroviride* TRS43 and *T. harzianum* TRS85—up to 30.00 and 9.85%, respectively (Fig. 2), the number of viable cells remained at high level, up to 10^7^ cfu/g (Table 1). The weakest storage stability was registered for *T. atroviride* TRS25 and for both tested *T. virens* strains (Fig. 2). After 3 months of storage, a general increase in the moisture content and water activity was observed; however, little change in pH value was registered (Table 2).

### The evaluation of enzymatic activities of *Trichoderma* strains in stored lyophilizates

**Fig. 3:**
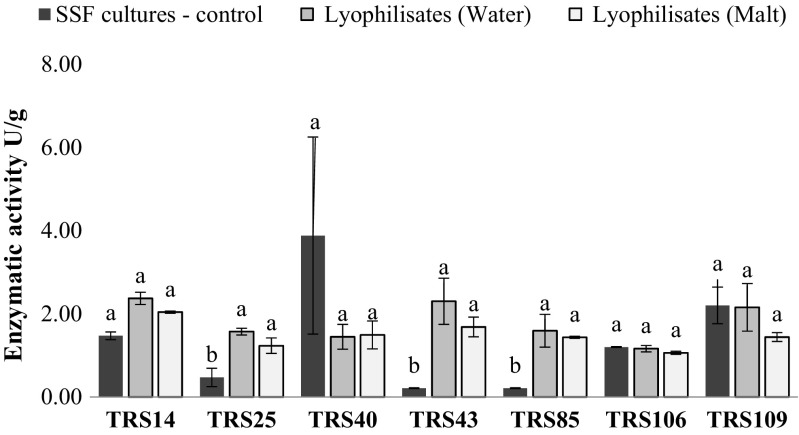
Cellulase activity of all tested *Trichoderma* strains after SSF cultures (before lyophilization process) and in the stored lyophilizates, with the addition of maltodextrin (malt) or distilled water (water). Values followed by different letters are significantly different according to LSD Fisher’s test (*p* < 0.05)

**Fig. 4:**
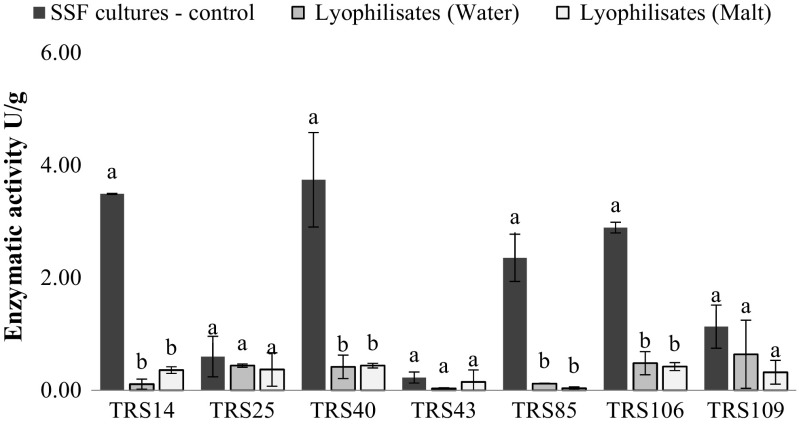
Polygalacturonase activity of all tested *Trichoderma* strains after SSF cultures (before lyophilization process) and in the stored lyophilizates, with the addition of maltodextrin (malt) or distilled water (water). Values followed by different letters are significantly different according to LSD Fisher’s test (*p* < 0.05)

**Fig. 5:**
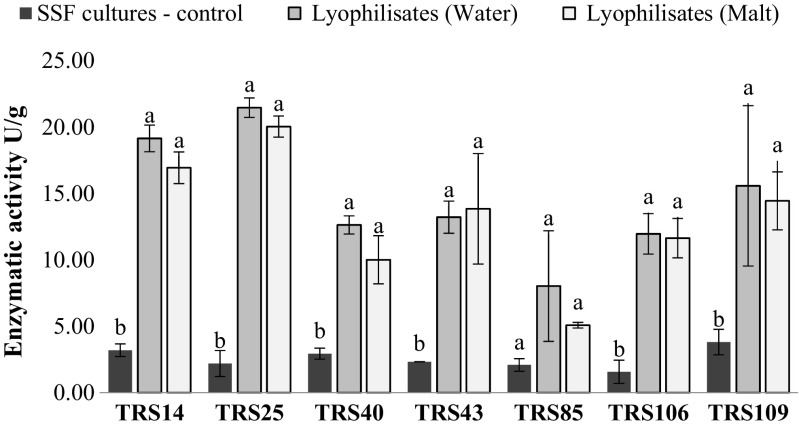
Xylanase activity of all tested *Trichoderma* strains after SSF cultures (before lyophilization process) and in the stored lyophilizates, with the addition of maltodextrin (malt) or distilled water (water). Values followed by different letters are significantly different according to LSD Fisher’s test (*p* < 0.05)

In control cultures, inoculated with fresh (non-preserved) conidial suspensions, *Trichoderma* strains were capable to synthetize extracellular hydrolytic enzymes at different levels, depending on the strain; however, the activity of these enzymes did not exceed 4 U/g for any of the tested strain. The highest activities of cellulases, and polygalacturonases in control SSF cultures, were registered for *T. atroviride* TRS40 (Figs. 3 and 4), whereas the highest level of xylanases was observed for *T. virens* TRS109 (Fig. 5).

After a 3-month storage of *Trichoderma* lyophilizates, the capability of tested strains to synthetize hydrolytic enzymes was evaluated. The cultures were individually inoculated with lyophilizates after 3 months of their storage at room temperature. The study showed that all tested strains maintained the capability for biosynthesis of cellulases and xylanases to higher extend than polygalacturonases. The presence of cryoprotectant—10% solution of maltodextrin in lyophilizates—had no significant effect on enzymatic activity of the tested strains (Figs. 3, 4, and 5). In general, cellulolytic and xylanolytic activities exhibited by most of the tested *Trichoderma* strains were of higher value in SSF cultures inoculated with freeze-dried conidia in comparison to the activities obtained in control SSF cultures, inoculated with fresh conidial suspensions (Figs. 3 and 5). In contrast, an inverse dependency was observed for polygalacturonases, where reduced activity of these enzymes was registered in SSF cultures inoculated with the freeze-dried conidia in comparison to control cultures (Fig. 4). The highest cellulolytic activity in SSF cultures, inoculated with lyophilizates, was observed for two strains of *T. atroviride*: TRS14 and TRS43 (Fig. 3). In the case of xylanases, the highest activity of these enzymes was also recorded for the strains of the same species: TRS25 and TRS14, and the enzymatic activity was almost sixfold and tenfold higher, respectively, as compared to activity of xylanases obtained in control SSF cultures (Fig. 5). Pectinolytic activity remained on a quite low level for all tested *Trichoderma* strains; the highest value was registered for *T. virens* TRS109 (Fig. 4).

## Discussion

The study aimed at the evaluation of *Trichoderma* strains in terms of their survivability and capability to synthesize hydrolytic enzymes after preservation process and after the storage of bioformulations. The choice of lignocellulosic components for SSF cultivation of fungal strains was based on the knowledge of their enzymatic capacity (Kovacs et al. 2009). Microscopic fungi, depending on their enzymatic activity, are able to decompose lignin, cellulose, and xylan in various ratios and at different polymer degradation levels. High capabilities of exocellular hydrolases production on lignocellulosic waste materials were reported for *Trichoderma reesei*, *T. longibrachiatum*, and *T. harzianum* (Mitchell et al. 2002; Juhasz et al. 2004; Sukumaran et al. 2005). These reports, along with the fact that lignocellulosic wastes are abundantly generated by agro-industry sectors, such as among others, cereal production, paper, and textile industry, prompted authors to use them as SSF medium component. The use of wheat straw in SSF for the production of fungal biomass and conidia, subsequently subjected to lyophilization, was also dictated by our previous studies (data not shown), where *T. harzianum* and *T. atroviride* strains gave satisfactory results in biomass yield in SSF biomass production on wheat straw. Tewari and Bhanu (2004) obtained the yield of *T. harzianum* of 2.65 × 10^8^ cfu/g of dry mass in SSF on wheat straw, whereas in our studies, *T. harzianum* TRS85 showed almost twice as high biomass yield in the SSF cultures on wheat straw, using similar culturing conditions. Additionally, wheat straw may have a protective effect on conidia during lyophilization after the SSF cultures (Tewari and Bhanu 2004; Kancelista and Witkowska 2008).

In the present study, the number of viable cells after freeze-drying process ranged from 10^7^ to 10^8^ cfu/g among the species of *T. atroviride* and *T. harzianum*, whereas for *T. virens*, it ranged from 10^5^ to 10^6^ cfu/g. In their studies, Kumar et al. (2014) claimed that the number of colony-forming units in biopreparations, which determines the effectiveness of *Trichoderma* sp., is 2 × 10^6^ cfu/g. In presented research, most of the tested strains met the criterion proposed by these authors. In general, the addition of maltodextrin as cryoprotectant had no significant effect on survivability of tested *Trichoderma* strains. Diversely, maltodextrin affected significantly the storage stability of two *T. atroviride* strains, TRS14 and TRS40, where it was higher as compared to control lyophilizates. For other tested strains, the addition of cryoprotectant had no effect on storage stability of *Trichoderma* strains. Panahian et al. (2012) subjected the biomass of *Trichoderma*, produced in SSF cultures on molasses and corn grains to freeze-drying process, while testing cryoprotectants, including maltodextrin, and they did not registered its beneficial effect on the survival of lyophilized strains. In turn, Sargin et al. (2013) reported, after freeze-drying process of *T. harzianum* on wheat straw and barley malt (3:2), the survival of 29%, whereas in our studies the survivability of *T. harzianum* TRS85 lyophilized on wheat straw was in the range of 79–100%. In presented study, the storage stability of *Trichoderma* strains in lyophilizates was also monitored after 3 months of their storage at room temperature and it was shaped in a very broad range from approximately 0.08 to 95%. Panahian et al. (2012) noted a reduction in the survival rate after the 3-month storage of freeze-dried *T. atroviride* and *T. koningii* strains, where it was approximately 9% in relation to the control, i.e., immediately after the freeze-drying process. Although the storage stability varied among strains tested in this study, all *T. atroviride* strains and *T. harzianum* exhibited considerable number of viable cells after the 3-month storage of lyophilizates, which ranged from 10^6^ to 10^7^ cfu/g. According to literature data, the survivability of preserved *Trichoderma* strains depends on many factors. Bhat et al. (2009) reported 10^5^ to 10^8^ cfu/g for *T. harzianum* strain, preserved on talc with the addition of carboxymethylcellulose after a 6-month storage of biopreparations. Similarly, Gade et al. (2009) investigated the storage stability of *T. harzianum* and *T. virens* in biopreparations added with talc and stored for 6 months, achieving 10^7^ cfu/g, where in turn, Singh et al. (2007) reported low number of viable cells of *T. harzianum* that ranged from 10^4^ to 10^5^ cfu/g after 7 months of storage on different carriers, such as bananas, corn cobs and flour, wheat bran, saw dust, and sorghum grain, which are considered rich and highly diversified components for culture media. In our previous study (Witkowska et al. 2016), we noticed that *T. atroviride* TRS40 survivability after preservation by fluidized bed drying at various temperatures ranged from 40 to 100%. These results might indicate that, apart from preservation method, type of a carrier, and addition of protectants, the stability of conidia in biopreparations are also affected by physicochemical parameters. In present study, water activity and pH values were determined after storage of *Trichoderma* lyophilizates. Longa et al. (2008) reported that *T. atroviride* growth is inhibited in pH values above 8.0. In our research, pH values did not change significantly after the 3-month storage of lyophilizates and remained slightly alkaline; hence, all tested *T. atroviride* strains exhibited high number of viable cells. The optimal *a*_w_ value to retain high stability of conidia of *T. harzianum* stored at room temperature was reported as 0.32 (Fernández-Sandoval et al. 2012). In presented study, the *a*_w_ did not exceed 0.24, which we consider a desirable water activity for preserved bioformulations.

Biopreparations should exhibit both high survivability and storage stability, as well as the capability for biosynthesis of hydrolytic enzymes after preservation process and storage. Therefore, we also evaluated the levels of production of cellulases, xylanases, and polygalacturonases of *Trichoderma* strains in lyophilizates stored for 3 months at room temperature. In the present study, the level of production of xylanases in SSF cultures, inoculated with lyophilizates of *Trichoderma*, was in general higher than in the control cultures. The synthesis of enzymes, especially hydrolases, is most commonly induced by the substrate, which is associated with the launch of the entire genetic material (which is associated with the processes of the following: replication, transcription, and translation). We speculate that, after storage of biomass and conidia on the lignocellulosic material, the reinforced biosynthesis of enzymes might have occurred caused by changes in the cells influenced by lignocellulosic components of media, resulting in higher enzymes activity after storage of freeze-dried bioformulations than prior to lyophilization process. Based on these speculations and the data obtained in this study, the increased xylanase activity in SSF cultures, inoculated with freeze-dried conidia, might have occurred due to specific enhancement of the biosynthesis of these enzymes induced by the presence of wheat straw, consisting mainly of hemicellulose, in the culture medium. Additionally, the pH optimum for the xylanolytic activity of *Trichoderma* is in the range of 3.5–6.0, which is a wider range than in case of cellulases (Tokarzewska-Zadora et al. 2005). This might explain the fact that in the present study and in the studies conducted by Kancelista and Witkowska (2008), higher levels of xylanases than cellulases were obtained. Moreover, the carbon source in the culture SSF may affect biosynthesis of xylanases by *Trichoderma.* Soliman et al. (2012) recorded the biosynthesis of xylanases by *T. viride* in SSF cultures, with the addition of barley bran and wheat straw. Also, in presented study, an increased activity of xylanases was recorded in SSF cultures with wheat straw. The ability to biosynthesis of polygalacturonases by *Trichoderma* strains used in this study, retained low level after 3 months of storage of lyophilizates. According to Shuster and Shmoll (2010), optimum pH for the biosynthesis of polygalacturonases by *Trichoderma* is in the range of 4.0–5.0, and the pH of SSF cultures, carried out in this study, was slightly alkaline. Also, wheat straw does not contain a large amount of pectin; therefore, *Trichoderma* strains were not stimulated for the production of polygalacturonases, involved in its decomposition. Zuoxing and Kalidas (2000) observed that the supplementation of SSF culture medium with apple pomace increased the production of polygalacturonases by another filamentous fungus of species *Lentius edodes.* Moreover, according to Mohamed et al. (2003) and Olsson et al. (2003), the activity of these enzymes significantly decreases outside the abovementioned pH range. These observations may explain the low activity of polygalacturonases obtained in this study. However, in literature, there are reports about high levels of xylanase and polygalacturonase biosynthesis by *Trichoderma*, in media supplemented with peels of citrus fruits (watermelons, bananas, oranges), in cultures carried out at pH 6.0–7.0, 50–66% humidity, and a temperature of 28–35 °C (Mohamed et al. 2013). Therefore, it might be assumed that the presence of the substrate rich in pectin is a more important factor in the synthesis of these enzymes than pH values and the temperature of the culture.

Based on literature reports and on presented results, it can be concluded that the resistance of conidia and biomass of *Trichoderma* to the process of freeze-drying and the survival rate of cells, along with the ability to biosynthesize hydrolytic enzymes, depend on biotic and abiotic factors, and the high storage stability may not be necessarily correlated with the high biosynthesis of extracellular enzymes. Because of the high demand for enzymes of microbial origin, such as cellulases, xylanases, and polygalacturonases for the lignocellulosic waste management from agriculture and a variety of industries, *Trichoderma* strains and other microorganisms are often subjected to biotechnological methods, providing the enhancement of enzymatic activities. This work gives an insight into the use of wheat straw for the production of biomass, conidia and enzymes on considerable levels in SSF cultures, as well as into the use of this agriculture waste as carrier and protectant during freeze-drying process and storage of biopreparations of *Trichoderma*.

## References

[CR1] Bai Z, Jin B, Li Y, Chen J, Li Z (2008). Utilization of winery wastes for *Trichoderma viride* biocontrol agent production by solid state fermentation. J Environ Sci.

[CR2] Benítez T, Rincón AM, Limón MC, Codón AC (2004). Biocontrol mechanisms of *Trichoderma* strains. Int Microbiol.

[CR3] Bhat KA, Anwar A, Lone GM, Hussain K, Nazir G (2009). Shelf life of liquid fermented product of *Trichoderma harzianum* in talc. J Mycol Pl Pathol.

[CR4] Daryaei A, Jones EE, Glare TR, Falloon RE (2016). Biological fitness of *Trichoderma atroviride* during long-term storage, after production in different culture conditions. Biocontrol Sci Techn.

[CR5] Day J, Stacey G (2007). Cryopreservation and freeze-drying protocols.

[CR6] Druzhinina IS, Komoń-Zelazowska M, Atanasova L, Seidl V, Kubicek CP (2010). Evolution and ecophysiology of the industrial producer *Hypocrea jecorina* (anamorph *Trichoderma reesei*) and a new sympatric agamospecies related to it. PLoS One.

[CR7] Fernández-Sandoval MT, Ortiz-Garcia M, Galindo E, Serrano-Correon L (2012). Cellular damage during drying and storage of *Trichoderma harzianum* spores. Process Biochem.

[CR8] Gade RM, Wardhe SR, Armarker SV (2009). Shelf life study of *Trichoderma* spp. in different carrier materials. Journal of Maharashtra Agricultural Universities.

[CR9] Ganga A, Gonzalez-Candelas L, Ramon D, Perez-Gonzalez JA (1997). Glucose-tolerant expression of *Trichoderma longibrachiatum* endoglucanase I, an enzyme suitable for use in wine production. J Agric Food Chem.

[CR10] Grzegorczyk M, Szalewicz A, Żarowska B, Połomska X, Wątorek W, Wojtatowicz M (2015). Microorganisms in biological control of phytopathogenic fungi. Acta Sci Pol Biotechnol.

[CR11] Gusakov AV (2011). Alternatives to *Trichoderma reesei* in biofuel production. Trends Biotechnol.

[CR12] Juhasz T, Szengyel Z, Reczey K, Siika-Aho M, Viikari L (2004). Characterization of cellulases and hemicellulases produced by *Trichoderma reesei* on various carbon sources. Process Biochem.

[CR13] Kancelista A, Witkowska D (2008). Biosynthesis of some lytic enzymes in medium containing waste corn cobs by filamentous fungi from *Trichoderma* genus. Acta Sci Pol Biotechnol.

[CR14] Kancelista A, Tril U, Stempniewicz R, Piegza M, Szczech M, Witkowska D (2013). Application of lignocellulosic waste materials for the production and stabilization of *Trichoderma* biomass. Pol J Environ Stud.

[CR15] Kovacs K, Macrelli S, Szakacs G, Zacchi G (2009). Enzymatic hydrolysis of steam-pretreated linocellulosic materials with *Trichoderma atroviride* enzymes produced in-house. Biotechnol Biofuels.

[CR16] Kuhad RC, Gupta R, Singh A (2011). Microbial cellulases and their industrial applications. Enzyme Res.

[CR17] Kumar S, Thakur M, Rani A (2014). *Trichoderma*: mass production, formulation, quality control, delivery and its scope in commercialization in India for the management of plant diseases. Afr J Agric Res.

[CR18] Longa CMO, Pertot I, Tosi S (2008). Ecophysiological requirements and survival of *Trichoderma atroviride* isolate with biocontrol potential. J Basic Microbiol.

[CR19] Magan N (2001). Physiological approaches to improving the ecological fitness of fungal biocontrol agents, fungi as biocontrol agents.

[CR20] Masutti DC, Borgognone A, Setti L (2012) Production of enzymes from rice husks and wheat straw in solid state fermentation. 3rd International Conference on Industrial Biotechnology (IBIC), Vol: 27. doi: 10.3303/CET1227023

[CR21] Miller GL (1959). Use dinitrosalicylic acid reagent for determination of reducing sugars. Anal Chem.

[CR22] Mitchell DA, Berovic M, Krieger N (2002). Overview of solid state bioprocessing. Biotechnol Annu Rev.

[CR23] Mohamed S, Christensen T, Mikkelsen J (2003). New polygalacturonases from *Trichoderma reesei*: characterization and their specificities to partially methylated and acetylated pectins. Carbohydr Res.

[CR24] Mohamed SA, Al-Malki AL, Khan JA, Kabli SA, Al-Garni SM (2013). Solid state production of polygalacturonase and xylanase by *Trichoderma* species using cantaloupe and watermelon rinds. J Microbiol.

[CR25] Olsson L, Christensen T, Hansen K, Palmqvisit E (2003). Influence of the carbon source on production of cellulases, hemicellulases and pectinases by *Trichoderma reesei* Rut C-30. Enzyme Microb Tech.

[CR26] Panahian R, Rahnama K, Jafari M (2012). Mass production of *Trichoderma* ssp. and application. Intl Res J Appl Basic Sci.

[CR27] Pandey A (1991). Aspects of fermenter design for solid-state fermentations. Process Biochem.

[CR28] Pandya JR, Sabalpara AN, Chawda SK (2011) *Trichoderma*: a particular weapon for biological control of pathogens. J Agric Technol 7(5):1187–1191

[CR29] Phitsuwan P, Laohakunjit N, Kerdchoechuen O, Kyu KL, Ratanakhanokchai K (2013). Present and potential applications of cellulases in agriculture, biotechnology, and bioenergy. Folia Microbiol.

[CR30] Piegza M, Stolaś J, Kancelista A, Witkowska D (2009). Influence of *Trichoderma* strains on the growth of pathogenic moulds in biotic test on untypical carbon sources. Acta Sci Pol Biotechnol.

[CR31] Sanghvi GV, Koyani RD, Rajput KS (2010). Thermostable xylanase production and partial purification by solid-state fermentation using agricultural waste wheat straw. Mycology.

[CR32] Sargin S, Gezgin Y, Eltem R, Vardar F (2013). Micropropagule production from *Trichoderma harzianum* EGE-K38 using solid-state fermentation and a comparative study for drying methods. Turk J Biotechnol.

[CR33] Shuster A, Shmoll M (2010). Biology and biotechnology of *Trichoderma*. Appl Microbiol Biot.

[CR34] Singh A, Srivastava S, Singh HB (2007). Effect of substrates on growth and shelf life of *Trichoderma harzianum* and its use in biocontrol of diseases. Bioresour Technol.

[CR35] Skoneczny D, Oskiera M, Szczech M, Bartoszewski G (2015). Genetic diversity of *Trichoderma atroviride* strains collected in Poland and identification of loci useful in detection of within species diversity. Folia Microbiol.

[CR36] Smolińska U, Kowalska B, Kowalczyk W, Szczech M (2014). The use of agro-industrial wastes as carriers of *Trichoderma* fungi in the parsley cultivation. Sci Hortic.

[CR37] Soliman HM, Abdel-Dayem A, El-Tanash AB, El-Tanash SA (2012). Production of xylanase by *Aspergillus niger* and *Trichoderma viride* using some agricultural residues. Int J Agric Res.

[CR38] Sukumaran RK, Singhania RR, Pandey A (2005) Microbial cellulases - Production, applications and challenges. J Sci Ind Res 64:832–844

[CR39] Sukumaran RK, Singhania RR, Mathew GM, Pandey A (2009). Cellulase production using biomass feed stock and its application in lignocellulose saccharification for bio-ethanol production. Renew Energy.

[CR40] Szczech M, Staniaszek M, Habdas H, Uliński Z, Szymański J (2008). *Trichoderma* spp.—the cause of green mold on Polish mushroom farms. Vegetable Crops Research Bulletin.

[CR41] Szczech M, Witkowska D, Kancelista A, Piegza M, Gajewska E, Małolepsza U (2011) Method for selection of active fungal isolates of the genus *Trichoderma*. Patent Application No. PL397659. Dec 30, 2011

[CR42] Tan CS, van Ingen CW, Stalpers JA (2007). Freeze-drying fungi using a shelf freeze-drier. Methods Mol Biol.

[CR43] Tewari L, Bhanu C (2004). Evaluation of agro-industrial wastes for conidia based inoculums production of biocontrol agent: *Trichoderma harzianum*. J Sci Ind Res.

[CR44] Texido N, Canamas TP, Usall J, Torres R, Magan N, Vinas I (2005). Accumulation of the compatible solutes, glycine-betaine and ectoine, in osmotic stress adaptation and heat shock cross-protection in the biocontrol agent Pantoea agglomerans CPA-2. Lett App Microbiol.

[CR45] Tokarzewska-Zadora J, Rogalski J, Szczodrak J (2005). Xylan-degrading enzymes—characterization and application in biotechnology. Biotechnologia.

[CR46] Valmaseda M, Martínez MJ, Martínez AT (1991). Kinetics of wheat straw solid-state fermentation with *Trametes versicolor* and *Pleurotus ostreatus*—lignin and polysaccharide alteration and production of related enzymatic activities. Appl Microbiol Biotechnol.

[CR47] Witkowska D, Bień M (1991). Activity in biosynthesis of extracellular hydrolases of *Trichoderma viride* mutants obtained in two-stage mutation. Acta Alient Polon.

[CR48] Witkowska D, Maj A (2002). Production of lytic enzymes by *Trichoderma* spp. and their effect on the growth of phytopathogenic fungi. Folia Microbiol.

[CR49] Witkowska D, Wróblewska A, Jurgielewicz W (1997). Degradation of cellulose and lignocellulose by *Trichoderma reesei* M7-1 hydrolases. Pol J Food Nutr Sci.

[CR50] Witkowska D, Kancelista A, Wilczak A, Stempniewicz R, Pasławska M, Piegza M, Łaba W, Szczech M (2016). Survivability and storage stability of *Trichoderma atroviride* TRS40 preserved by fluidised bed drying on various agriculture by-products. Biocontrol Sci Tech.

[CR51] Zuoxing Z, Kalidas S (2000). Solid state production of polygalacturonase by *Lentinusedodes* using fruit processing wastes. Process Biochem.

